# The ability of a submaximal cycle ergometer test to detect longitudinal changes in VO_2_max

**DOI:** 10.1186/s13102-021-00387-w

**Published:** 2021-12-14

**Authors:** Frida Björkman, Örjan Ekblom, Elin Ekblom-Bak, Tony Bohman

**Affiliations:** 1 Department of Physical Activity and Health, The Swedish School of Sport and Health Sciences, P.O Box 5626, 114 86 Stockholm, Sweden; 2 School of Health and Welfare, Dalarna University, 791 88 Falun, Sweden

**Keywords:** Cycle ergometer test, Estimation, Fitness, Maximal oxygen uptake

## Abstract

**Background:**

The purpose of the present study was to examine the ability of a submaximal cycling test to detect longitudinal changes in maximal oxygen uptake (VO_2_max) and examine the conformity between changes in measured and estimated VO_2_max over a time span of 5–8 years.

**Methods:**

A total of 35 participants (21 men and 14 women), aged 29 to 63 years, performed the Ekblom-Bak (EB) submaximal cycle test for estimation of VO_2_max and a maximal treadmill running test for direct measurement of VO_2_max. The baseline tests were conducted between 2009 and 2012, and the follow-up tests were completed 5 to 8 years later. Pearson’s coefficient of correlation (*r*) and paired sample *t*-test were used to analyse the association between change in measured and estimated VO_2_max. Random and systematic errors between the measured and estimated VO_2_max were evaluated using Bland-Altman plots. Repeated measures ANOVA were used to test differences between changes over time.

**Results:**

There was no significant change in mean measured VO_2_max between baseline and follow-up (*p* = 0.91), however large individual variations were noted (− 0.78 to 0.61 L/min). The correlation between individual change in measured and estimated VO_2_max was *r* = 0.75 (*p* < 0.05), and the unstandardised B-coefficient from linear regression modelling was 0.88 (95% CI 0.61 to 1.15), i.e., for each litre of change in estimated VO_2_max, the measured value had changed 0.88 L. The correlation between baseline and follow-up errors (the difference between estimated-measured VO_2_max at each occasion) was *r* = 0.84 (*p* < 0.05). With regard to the testing procedure, repeated measures ANOVA revealed that there was no significant difference between the group who exercised at the same work rates at baseline and follow-up (n = 25), and those who required a change in work rate (n = 10).

**Conclusions:**

The EB test detected a change in VO_2_max with reasonably good precision over a time span of 5–8 years. Further studies are needed to evaluate if the test can be used in clinical populations and in subjects with different medications.

## Background

Cardiorespiratory fitness is an expression of the capacity of the pulmonary and circulatory systems and can be assessed through measurement of maximal oxygen consumption (VO_2_max). VO_2_max is the highest possible rate of oxygen consumption during a physically intense whole-body activity, and an important factor for many types of physical performance. It has also been linked to several health-related outcomes, with a low capacity being strongly associated with increased risk for cardiovascular disease, the metabolic syndrome, a negative influence on cognitive function, and more recently, severe Covid-19 [1–5]. In a scientific statement from the American Heart Association, the inclusion of measurement of VO_2_max in clinical practice is urged for to provide the clinicians with vital possibilities to improve patient risk management and health [6]. Though, golden standard for VO_2_max assessment includes direct measurements of VO_2_max performed with expensive laboratory equipment and require a maximal physical effort from the subject. This limits the assessment of VO_2_max in the general population or in large scale settings, and therefore submaximal exercise tests are commonly used. Many of these submaximal tests are executed on cycle ergometers [7–10].

One of the first and most popular submaximal cycle test is the Åstrand test, where the prediction of VO_2_max is based on the steady-state heart rate (HR) response to one single work rate [9]. More recently, the Ekblom-Bak test (EB test) was presented, which estimates VO_2_max from the relation between change in HR (∆HR) in response to an increase in work rate (power output, PO) from a low, standard work rate to a higher, individually chosen work rate (ΔHR/ΔPO) [10]. The estimation of VO_2_max from the revised EB test is derived from sex-specific prediction equations, which include age, ∆HR/∆PO, ∆PO, and HR at standard work rate as independent variables [11].

Not only the absolute level of VO_2_max, but also longitudinal intra-individual change in VO_2_max, is highly relevant to assess. For example, a decline in VO_2_max over time has been linked to incident hypertension [12], stroke [13] and dementia [14]. The importance of assessing cardiorespiratory fitness in clinical practice is well known [6], and the possibility to evaluate the effect of training programme in general population or in athletes is highly relevant for motivation and performance. However, it is largely unknown to what extent longitudinal changes in VO_2_max can be accurately detected via submaximal cycle ergometer tests. A rather limited ability to capture long-term changes in cardiorespiratory fitness by the Åstrand test was reported in a 20-year follow-up study of cardiorespiratory fitness in healthy adults [15]. In children, an enhanced VO_2_max was indicated with the Åstrand test after a training period, even though no actual change in measured VO_2_max was present [16].

The use of the EB test to detect changes in VO_2_max over time has not yet been evaluated. Therefore, the aim of the present study was to examine the ability of a submaximal cycling test to detect longitudinal changes in VO_2_max by evaluating the relation between the measured and estimated changes in VO_2_max over a period of 5 to 8 years.

## Methods

The subjects were recruited based on records from a previous study, conducted between year 2009 and 2012 (baseline) [10]. The aim of that study was to develop the EB test and its associated prediction equation in a mixed population regarding sex, age, and fitness status. The main inclusion criteria for participating in the present study (follow-up) was the availability of complete data from the baseline study on at least one valid submaximal cycle test and one valid measurement of VO_2_max from a maximal treadmill test. Exclusion criteria were smoking, use of snuff, and medication with β-stimulating or β-blocking agents. At follow-up in 2017, a total of 38 subjects, fulfilling the inclusion criteria, visited the Åstrand laboratory at the Swedish School of Sports and Health Sciences (Stockholm, Sweden). Test preparations as well as tests were performed at the same way in the present follow-up study as in the previous baseline study. The participants were requested to refrain from vigorous physical activity the day before the test and on the test day, and to not consume a heavy meal less than 3 h before the test. All subjects were fully informed of the study details before the first visit at the laboratory and provided written informed consent before start of the tests. At follow-up, one of the volunteers was excluded due to use of β-blocking agents, one subject failed to achieve a valid VO_2_max measurement (see criteria below), and one was diagnosed with a serious disease, giving a total of 35 included subjects.

Height and body mass were measured to the nearest 0.1 cm and 0.1 kg, respectively, with the participants wearing light clothing. Resting blood pressure and HR were measured with conventional methods after 10 min of supine rest. The physical tests comprised of a submaximal EB test and a maximal treadmill test (described in detail below). All recordings of HR were performed with the HR monitor RS400 (Polar Electro, Kempele, Finland). The respiratory breath-by-breath measurements were made with the computerised metabolic system Oxycon Pro (Jaeger, Hoechberg, Germany). HR and ventilatory variables were measured continuously during the physical tests. Before each test, ambient temperature, humidity, and barometric pressure were measured. The inspiratory flowmeter and gas analysers were calibrated with the built-in, automated calibration procedures, including control of low and high flow and gas analyse calibration with high-precision gases (15.00 ± 0.01% O_2_ and 6.00 ± 0.01% CO_2_, Air Liquide, Kungsängen, Sweden) and ambient indoor air. The study was approved by the Ethical Review Board in Stockholm, ref. no. 2016/175-31/2.

### Submaximal cycle test

All submaximal tests in the previous baseline and the present follow-up study were conducted on a Monark cycle ergometer model 828E (Monark Exercise AB, Vansbro, Sweden). All ergometers are carefully calibrated at the factory [17], and the calibration is regularly checked by experienced staff at the laboratory. Zeroing of the scale was performed while the subject sat on the saddle with the feet on the frame between the pedals and hands on the handlebars. The saddle was set to the same height at baseline and follow-up tests, and the handlebar was individually adjusted to each subject. Thereafter, the subject was verbally and visually introduced to the Borg RPE scale® for ratings of perceived exertion (RPE) [18]. The lowest and the highest rating of the scale is 6 and 20, representing no exertion and maximal exertion. The RPE scale® along with written instructions had also been sent to all participants before the first visit at the laboratory. The instructions state that the subject should rate the overall exertion, i.e., try to combine the central exertion as well as the exertion in legs/arms. Before each test, all subjects were asked to rate the current RPE for resting or sitting to verify their understanding of the scale. The participants then performed an EB test with a constant pedal frequency of 60 revolutions per min (rpm). The protocol of the EB test is described in detail elsewhere [10, 11]. The test started with 4 min of cycling at the standard work rate with a resistance of 0.5 kilopond (kp), ~ 30 watts (W). The second submaximal work rate was individually chosen to obtain an RPE of ~14 and the test was terminated if the subject rated a perceived exertion higher than 16 [10]. The selection of the higher work rate was based on the subjects self-reported physical status and training habits. HR and VO_2_ were recorded as the mean of the last minute for each work rate. The estimated VO_2_max were calculated using the sex-specific EB prediction equations, previously shown to have good validity and reliability in mixed populations [11, 19].

### Maximal test

The VO_2_max was measured during treadmill running. All subjects had a short rest after the cycle test, followed by an individually adjusted warm-up session on the treadmill. The warm-up session comprised of ~ 5–10 min running at a comfortable speed, followed by ~ 1 min of uphill running at an incline of 4–5°. Safe stopping technique was also explained to the subject. The procedure was practiced under controlled forms. The warm-up was followed by a brief rest (≤ 5 min), sometimes including intake of small amounts of water, some pre-conditioning stretching, or other individual preparation that the subject wished for. All maximal test protocols were individually designed. The tests started at 1° incline and the same speed as the subject felt comfortable with during the warm-up (a speed that corresponded to approximately 60–65% of estimated maximal capacity). Speed and/or inclination was increased every minute in steps of 1 km/h or 1°, until subjects reported maximal exhaustion. All test protocols were individually designed, and the workloads were selected to elicit a total exercise time of 5–8 min before exhaustion occurred. All researchers that conducted the tests had years of experience of maximal treadmill testing, and the changes in speed or inline were determined individually for each subject in order to reach as high VO_2_ as possible. All maximal tests were accompanied by extensive verbal encouragement. Criteria for inclusion of the VO_2_max measurement were a “levelling off” in VO_2_ despite an increase of speed or incline, with support of a respiratory exchange ratio (RER) ≥ 1.10, RPE ≥ 17, maximal HR within ± 15 beats per minute (bpm) from age-predicted maximum, and a work time ≥ 5 min. The test was included in the statistical analysis if levelling off was present in conjunction with at least 3 out of 4 of the other criteria. Determination of “levelling off” was done according to visual inspection of the VO_2_-curve with a clear levelling off or decline in VO_2_ despite increase in speed or velocity. VO_2_max was defined as the highest recorded oxygen uptake during 30 consecutive seconds, derived from the mean of the six highest consecutive 5 s epochs.

### Statistics

The assumption of normality was confirmed for all variables, using the skewness test for normality. The continuous descriptive characteristics were summarised as means with standard deviation (SD). Paired sample *t*-test was used to compare the subject characteristics and measured and estimated VO_2_max between the baseline and the follow-up test.

The Pearson’s coefficient of correlation (*r*), linear regression, and paired sample *t*-test were used to analyse the association between change in measured and estimated VO_2_max, and similarly to evaluate the association between the error in the EB test estimation (i.e., the difference between measured and estimated VO_2_max) from the baseline and the follow-up test.

Random and systematic errors between the measured and estimated VO_2_max were evaluated using Bland-Altman plots showing mean differences and limits of agreement (LoA) [20, 21]. Repeated measures ANOVA were used to test differences between changes over time. Additional analyses were made to evaluate the measurement error in subjects with changed or unchanged individual work rate, and increased or decreased VO_2_max, respectively. Furthermore, bivariate correlations were performed between change in measured VO_2_max and the individual variables in the EB test prediction equation.

Statistical significance was set at *p* < 0.05 and confidence intervals to 95% (95% CI) for all analyses. The magnitude of the correlation was interpreted using the recommendations suggested by Hinkle, where an *r* of 0.90 to 1.00 (− 0.90 to − 1.00) represents a very high correlation and 0.70 to 0.90 (− 0.70 to − 0.90) represents a high correlation [22]. The statistical analyses were conducted using IBM SPSS Statistics for Windows, version 24.0 (IBM Corp., Armonk, N.Y., USA).

## Results

Characteristics of the baseline and follow-up samples are presented in Table 1. In total, 21 men and 14 women were included. The mean time between tests was 6.4 years, ranging from 5.2 to 8.1 years. The females were 41.4 (10.2) years old at baseline, and 47.1 (10.6) years old at follow-up. The corresponding values for men were 43.7 (11.8) years old at baseline, and 49.3 (11.5) years old at follow-up. Women had a non-significant increase in mean body mass, from 61.3 to 62.4 kg (*p* = 0.08), ranging from − 3 kg to + 5.3 kg. Mean body mass among the men was almost unchanged, 81.0 kg at baseline and 80.8 kg at follow-up (*p* = 0.76), with individual changes ranging from − 4.5 to + 5.1 kg. The mean HR at the standard work rate of 30 W was unchanged between tests, however with large individual differences. Ten subjects had a decrease of 10 bpm or more (the greatest decrease was 23 bpm) and three subjects had an increase of ≥ 8 bpm (the greatest increase was 12 bpm) in the follow-up test compared to the baseline test. Maximal HR during test was significantly lower at follow-up, average drop of 3.8 bpm, with no significant difference between sexes (− 2.7 bpm for the women and − 4.5 bpm for the men, *p* = 0.05 and < 0.01, respectively). However, 10 of 38 subjects had an unchanged maximal HR (± 2 bpm), while in four cases it had dropped by as much as 10 to 13 bpm. Furthermore, four of the subjects had an increased maximal HR by 3 bpm.

**Table 1:** Subject characteristics at baseline test and follow-up test

	Baseline test	Follow-up test	n	*p*-value *
Age (years)	42.8 (11.1)	48.4 (11.0)	35	< 0.001
Height (cm)	177.0 (10.5)	176.8 (10.4)	35	0.21
Body Mass (kg)	73.1 (12.3)	73.5 (12.3)	35	0.48
Measured VO_2_max (L/min)	3.65 (0.91)	3.64 (0.89)	35	0.91
Measured VO_2_max (mL/kg/min)	49.7 (8.2)	49.5 (8.4)	35	0.76
Estimated VO_2_max (L/min)	3.56 (0.93)	3.47 (0.91)	35	0.05
Estimated VO_2_max (mL/kg/min)	48.5 (9.0)	47.2 (8.9)	35	0.04
VO_2_ at standard work rate (L/min)	0.82 (0.11)	0.81 (0.18)	26^a^	0.84
HR at standard work rate (bpm)	82.4 (12.2)	81.9 (11.7)	35	0.62
Maximal HR (bpm)	184.8 (14.6)	181.0 (14.4)	34^b^	< 0.001
ΔHR/ΔPO	0.41 (0.12)	0.39 (0.12)	35	0.08

All values are presented as means with standard deviation (SD)

VO_2_, Oxygen uptake; HR, heart rate; beats/min, beats per minute; ΔHR/ΔPO, change in heart rate/change in power output

*p-value for difference between baseline test and follow-up test

^a^Measurements of VO_2_ at standard work rate was conducted in a subsample of the baseline tests, and the value was missing for nine of the re-recruited subjects in the present study

^b^One subject had a missing maximal HR registration due to technical difficulties with the heart rate monitor during the final stages of the maximal running test

### Change in measured versus estimated VO_2_max

**Fig. 1:**
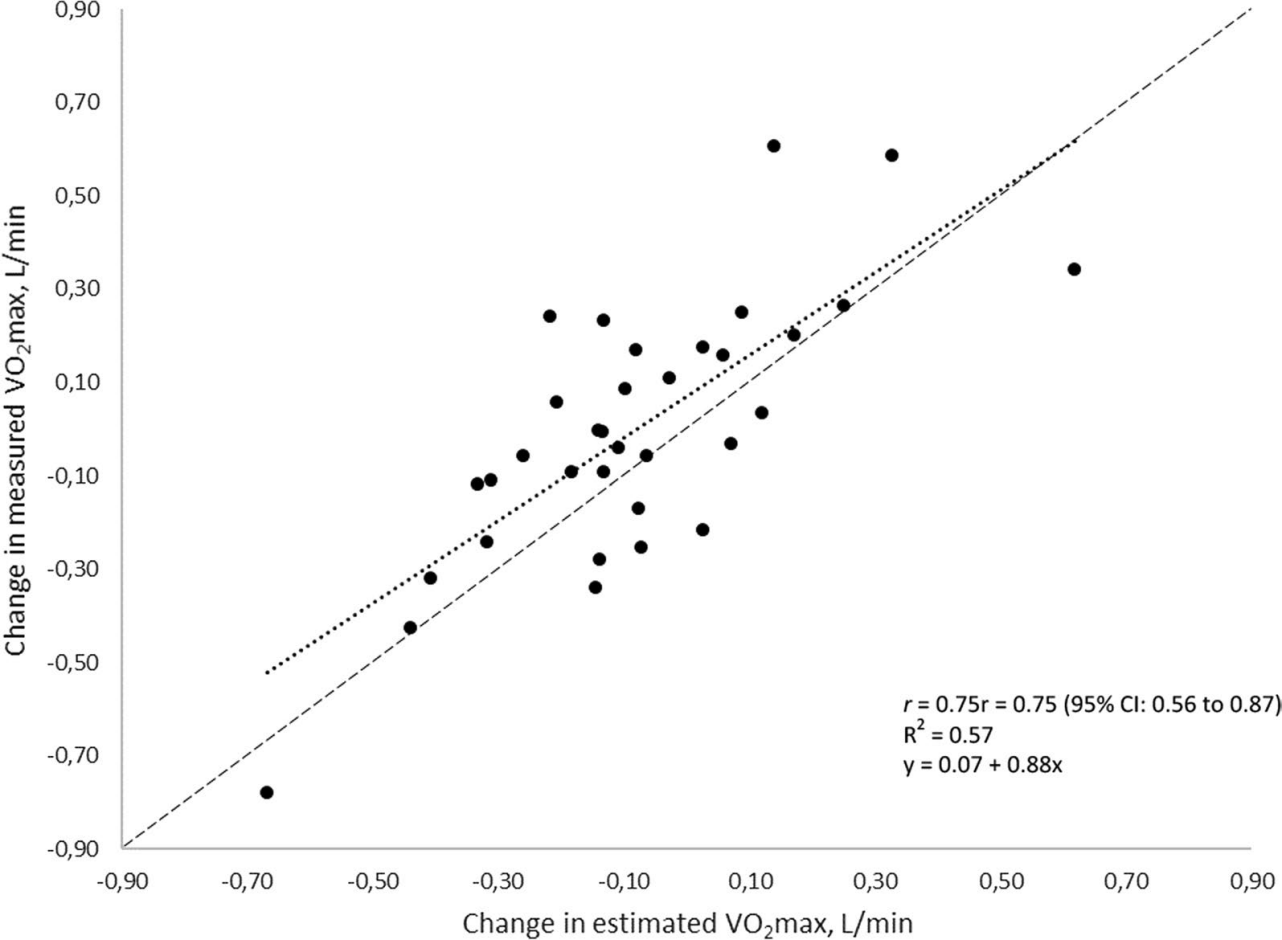
Change between the baseline test and the follow-up test in measured VO_2_max (y-axis) and estimated VO_2_max from the EB test (x-axis). Solid line: correlation. Dashed line: Line of identity. *r* = Pearson’s coefficient of correlation, R^2^ = coefficient of determination, 95% CI 95% confidence interval

There was a strong correlation between change in measured and change in estimated VO_2_max from baseline test to follow-up test (*r* = 0.75, *p* < 0.05), (Fig. 1). Linear regression analysis showed a significant unstandardised B-coefficient of 0.88 (95% CI: 0.61 to 1.15), indicating that for each litre of change in estimated VO_2_max, the measured value had changed 0.88 L. The largest individual decrease in measured and estimated VO_2_max was − 0.78 L/min and − 0.67 L/min, respectively. The corresponding values for the largest individual gain in measured and estimated VO_2_max was 0.61 L/min and 0.62 L/min, respectively. There was no significant change in mean measured VO_2_max from baseline to follow-up (− 0.01, 95% CI − 0.10 to 0.08), whereas the estimated VO_2_max was significantly lower at follow-up (− 0.08, 95% CI − 0.16 to − 0.00).

### Change in estimation error of VO_2_max

**Fig. 2:**
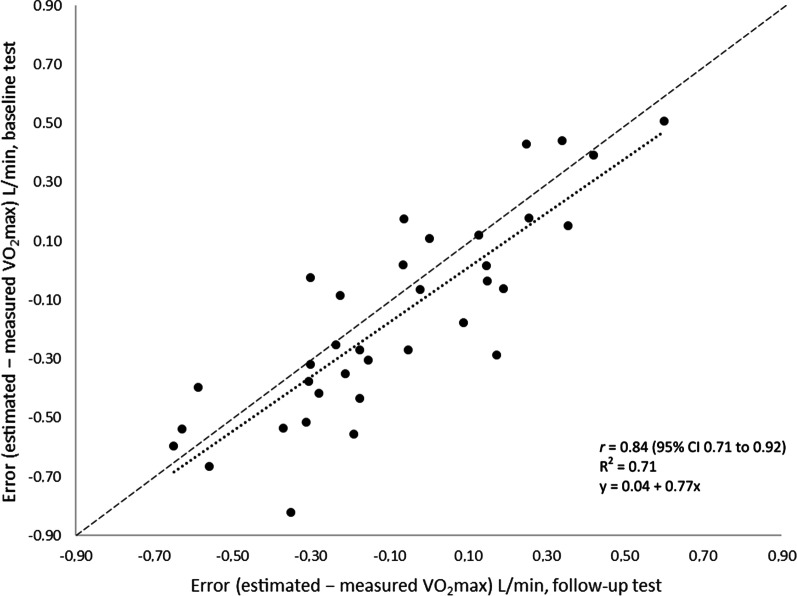
Error at the baseline test (y-axis) and error at the follow-up test (x-axis). Solid line: correlation. Dashed line: Line of identity. *r* = Pearson’s coefficient of correlation, R^2^ = coefficient of determination, 95% CI 95% confidence interval

There was a strong correlation between the estimation errors at the first test and at the follow-up test (Fig. 2), with a correlation of 0.84 (*p* < 0.05). When expressed as dichotomous change (i.e., increase in measured VO_2_max versus decrease in measured VO_2_max), subjects who increased measured VO_2_max had a significant change in estimation error (− 0.17 L/min, 95% CI − 0.24 to − 0.10, *p* = 0.01), while those who decreased measured VO_2_max had a small and non-significant change in estimation error (− 0.01 L/min, 95% CI − 0.06 to 0.04, *p* = 0.77).

**Fig. 3:**
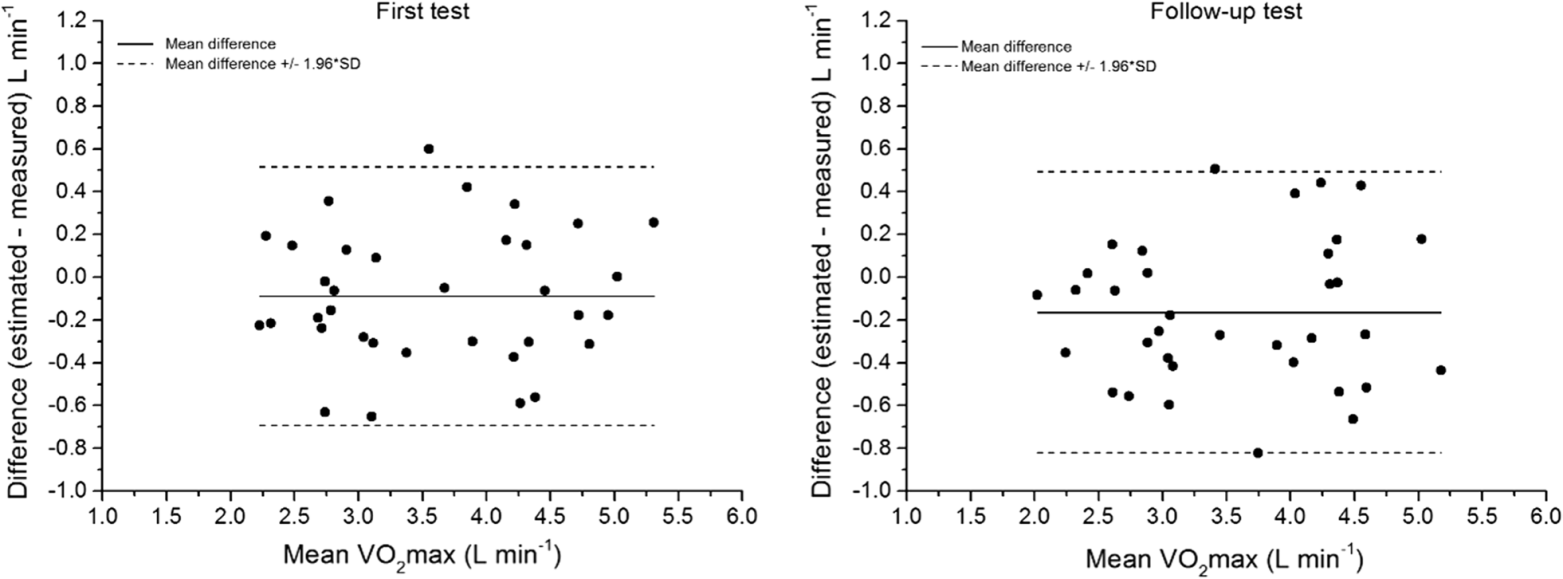
Bland Altman plots, including limits of agreement, for estimated and measured VO_2_max (L/min) at the baseline tests (left) and at the follow-up tests (right). Mean VO_2_max = (estimated + measured)/2. Black line: mean difference. Dashed line: ± 1.96 * SD

The overall mean difference between estimated and measured VO_2_max at baseline was − 0.09 (95% CI − 0.20 to 0.02) L/min at baseline and − 0.17 (95% CI − 0.29 to − 0.05) L/min at follow-up. The agreement of measurements is presented in the Bland-Altman plot (Fig. 3). The mean change in the error was − 0.08 L/min (LoA − 0.43 to 0.28 L/min). Thus, at the follow-up test, subjects were slightly more under-estimated than at the first test.

### Additional analyses

At the follow-up test, all subjects were asked about their current physical status in line with the recommended test procedure for the EB test and 71% of the subjects were evaluated to be capable of exercising at the same high work rate for the EB test as was used at baseline. Of the remaining subjects, 11% (4/35) cycled at a work rate that was 0.5 or 1.0 kp lower, and 17% (6/35) cycled at a work rate that was 0.5 or 1.0 kp higher than in the first test. The subjects who were tested at the same work rate displayed a significantly larger error at the follow-up test (mean error − 0.05 ± 0.31 L/min at the first test and − 0.15 ± 0.35 L/min at the second test, *p* = 0.01). The group with changes in the higher work rate displayed almost identical errors at both test occasions (baseline and follow-up errors of − 0.20 ± 0.30 L/min and − 0.20 ± 0.31 L/min, respectively, *p* = 0.87). However, repeated measures ANOVA revealed that there was no significant difference between groups. The mean HR for subjects that exercised on the same high work rate during both tests was significantly lower at follow-up, with values of 133.3 (14.6) bpm at baseline and 129.6 (13.9) bpm at follow-up. The corresponding values for the group with a different high work rate was 123.5 (18.8) at baseline and 123.2 (10.3) bpm at follow-up.

The bivariate correlations between change in measured VO_2_max and the individual variables in the EB test prediction equation showed that the change in ∆HR at standard work rate (*r* = − 0.42, *p* = 0.01) and change in ∆PO (*r* = 0.43, *p* = 0.01) were related to change in measured VO_2_max. Changes in the variables ∆HR/∆PO (*r* = − 0.27, *p* = 0.11) and age (*r* = − 0.23, *p* = 0.19) had no significant correlation to the change in measured VO_2_max.

## Discussion

The main finding of this study was that the EB test can be used to detect a change in VO_2_max over a time span of 5 to 8 years. There was a strong and significant correlation between changes in measured and changes in estimated VO_2_max values (*r* = 0.75, *p* < 0.05). Furthermore, the estimation errors (i.e., the difference between measured and estimated VO_2_max) seems to be rather constant over time (*r* = 0.84, *p* < 0.05). This means that anyone being overestimated at the first test, will also be so at the second test. As the measurement error (misclassification) was relatively constant over time, individual characteristics rather than temporary and/or environmental factors seem to contribute to the error.

Previous studies that have investigated whether longitudinal changes in VO_2_max can be accurately detected via submaximal cycle ergometer tests are scarce. In one study by Åstrand et al., all subjects demonstrated significantly lower values of measured VO_2_max in 1970 than in 1949, with a mean decrease of 22% in women and 20% in men. These changes was not possible to detect accurately with the submaximal Åstrand test [15]. The mean change in maximal HR was − 15 bpm for women and − 12 bpm for men, whereas some subjects had a rather unchanged value, and others displayed as much as 25–30 beats lower maximal HR. The mean HR at a given submaximal workload was higher at follow-up, but with large inter-individual differences. The explanation for these differences in submaximal HR responses could not be related to differences in VO_2_max (i.e., there was no correlation between decrease in maximal HR and decline in VO_2_max) or maximal HR (i.e., there was no correlation between change in maximal HR and HR at submaximal work rate. Consequently, it was not possible to accurately determine the change in measured VO_2_max through the submaximal Åstrand test and the associated nomograms [9, 23]. In contrast to the present study, those subjects were more homogenous regarding sex and physical fitness, and there was a considerably longer period between tests (the subjects were between 20 and 33 years old at baseline, and between 41 and 54 years old at follow-up).

The estimations of VO_2_max from submaximal exercise tests are often based on the rather linear relationship between HR and VO_2_, as well as HR and workload, with increasing exercise intensity. Cycling is a commonly used activity for submaximal exercise testing, since this work mode has relatively small inter-individual variations in work efficiency and because the mechanical efficiency is rather constant in mixed populations, even in populations that have never used or even seen a bicycle or cycle ergometer before [24]. Still, the assumption of a relatively static work efficiency (energy cost/VO_2_ consumption for produced W) has been questioned. It has been shown that higher body mass results in a higher energy consumption for a given rate of work [25]. In the previously mentioned study by Åstrand et al., the subjects displayed almost no differences in efficiency (measured as O_2_ uptake for a certain rate of work) in 1949 and 1970. The only exception from this observation was for the work rate of 200 W, where the men had a higher VO_2_ in 1970 compared to 1949 (2.84 L/min and 2.67 L/min, respectively). However, the sample comprised of healthy, physically fit and normal weight individuals, who had the same body mass on both test occasions. In a subsample in the present study, the VO_2_ uptake at the standard work rate was unchanged, and both changes in body mass and VO_2_ at the standard work rate were unrelated to the change in the estimation error, indicating similar work efficiency (data not shown).

The underpinning theory of HR-based exercise tests is often that a decrease in HR at a given submaximal workload is related to a positive training adaptation. A submaximal exercise test may be an efficient and easily accessible method to assess cardiac autonomic activity and to track changes in maximal aerobic capacity [26–28]. However, several factors contribute to the overall uncertainty in the interpretation of the HR response to a submaximal workload, and thereby contributes to the misclassification of VO_2_max from submaximal tests. Several of these factors are of extra importance for the measurement error in a test where the results are derived from the steady state HR at *one* single work rate, for example the Åstrand test. Environmental conditions, dehydration, ingestion of food and beverage and emotional stress may have an impact on a single HR measurement, as well may different medications alter the HR response to submaximal exercise. For example, treatment with chemotherapy induces autonomic dysfunctions [29] which may influence HR response to submaximal work, especially at lower levels of exercise [30]. Also in the normal population, it has been shown that intra-individual variations in submaximal HR is greater at lower exercise intensities than at higher intensities [31]. In contrast to the Åstrand test, the EB test is a two-point test (HR measurements at two work rates) with a prediction equation that includes the variable ΔHR/ΔPO (slope). This may counteract some of the measurement errors caused by the above-mentioned external factors and variations in HR response to a given rate of work, attenuating any misclassification at both a single test and at repeated tests.

The variations in VO_2_max over time can be due to naturally occurring factors such as the consequence of aging, as well as changes in exercise habits and other health-related aspects. Since submaximal tests generally are used in larger-scale settings (even epidemiological studies) it is important to study the ability to capture longitudinal changes (>5 years) in VO_2_max. However, this is not quite the same thing as experimentally increasing VO_2_max during a short period of intense aerobic training since the former type of study must take age-related changes into account. The ability of the test to detect training induced increases in VO_2_max was not investigated in the present study. Stroke volume increases as a consequence of aerobic training and the increased oxygen transport is the primary explanation to increased VO_2_max, so it is reasonable to hypothesise that the difference in HR between two work rates will be less pronounced. Furthermore, an altered arterio-venous O_2_ difference (avO_2_diff), either because of training or aging, may also alter the relation between VO_2_ and HR.

Regarding the choice of individual high work rate for repeated tests, *p*-values revealed a significant change in estimation error for the group that was tested with the same work rate at baseline and follow-up tests. However, no difference was found between groups with repeated measures ANOVA. These findings indicate that there is no need to keep the same higher work rate between tests to procure a good estimation. As part of the testing procedure, subjects were asked about their current physical status to select a correct higher work rate, resulting in both lowered and increased load compared to the baseline test. However, there was no significant difference between groups and there is no support to the recommendation to use the same individually chosen higher work rate when monitoring an individual over time. Every test and measurement can be assessed and judged for the specific test occasion. This approach is preferable with repeated tests over time (≥ 5 years), where the subject exhibits a change in physical capacity and consequently needs another individually chosen high work rate.

It has been suggested that the inclusion of cardiorespiratory fitness measurement in routine clinical practice is mandatory to provide an optimal approach for stratifying patients according to risk [6]. Submaximal exercise tests are highly important to provide VO_2_max values for risk prediction when direct measurements are not feasible. The development of valid and reliable submaximal cycle test, for example the EB test, has high relevance for coaches, researchers and clinicians. The present study showed that there was no significant change in the estimation error for subjects with a decrease in VO_2_max over time, which allows follow-up measurements to detect a decline in cardiorespiratory fitness and implementation of physical activity and training. However, evaluating long term changes in clinical populations or for training induced changes should be done with caution as this needs to be studied further.

This study has several limitations. The relatively small sample size made it impossible to conduct more detailed analyses in different subgroups, for example individuals with a pronounced change in body mass and/or body composition. It is important to examine the influence of these aspects since the test may be used in association with a weight loss- or training programme. The sample size may also limit the utilisation of BA-plots as a sample size of approximately 50 subjects is recommended in these analyses [32]. However, as this is not an exact requirement, and we have no extreme outliers in or sample, we believe our results to be reliable. A common issue in studies involving maximal testing is the selection bias, often including more fit individuals than in the general population. In the recruitment phase of the present study, individuals with a self-reported currently high training status were more willing to participate in the follow-up tests than those with a self-reported reduction in fitness level. The preponderance of well-trained subjects may eventually limit the accuracy of the prediction equation in the general population, where the normal physiological response to aging is a lowered VO_2_max.

## Conclusions

The EB test can well capture the actual changes in VO_2_max during a follow-up of 5 to 8 years. On an individual level, the misclassification in VO_2_max from the EB test is relatively constant over time. However, future studies are needed to assess the ability to detect training induced increases in VO_2_max as well as to evaluate if the test can be used to detect change in clinical populations, in subjects with different medications and in children and adolescents.

## Data Availability

The datasets used and/or analysed during the current study are available from the corresponding author on reasonable request.

## Abbreviations

ANOVA: Analysis of variances
bpm: Beats per minute
EB test: The Ekblom-Bak test
HR: Heart rate
kp: Kilopond
LoA: Limits of agreement
PO: Power output
RPE: Rate of perceived exertion
rpm: Revolutions per minute
VO_2_: Oxygen uptake
VO_2_max: Maximal oxygen uptake
ΔHR: The difference in HR between the high and low work rate
ΔHRmax: The change in maximal heart rate
ΔPO: The difference in work rate between the high and low work rate
